# B-Cells and Antibodies as Contributors to Effector Immune Responses in Tuberculosis

**DOI:** 10.3389/fimmu.2021.640168

**Published:** 2021-02-18

**Authors:** Willemijn F. Rijnink, Tom H.M. Ottenhoff, Simone A. Joosten

**Affiliations:** Department of Infectious Diseases, Leiden University Medical Center, Leiden, Netherlands

**Keywords:** *Mycobacterium tuberculosis*, tuberculosis, humoral immunity, B-cells, antibodies, biomarker

## Abstract

Tuberculosis (TB), caused by *Mycobacterium tuberculosis* (Mtb), is still a major threat to mankind, urgently requiring improved vaccination and therapeutic strategies to reduce TB-disease burden. Most present vaccination strategies mainly aim to induce cell-mediated immunity (CMI), yet a series of independent studies has shown that B-cells and antibodies (Abs) may contribute significantly to reduce the mycobacterial burden. Although early studies using B-cell knock out animals did not support a major role for B-cells, more recent studies have provided new evidence that B-cells and Abs can contribute significantly to host defense against Mtb. B-cells and Abs exist in many different functional subsets, each equipped with unique functional properties. In this review, we will summarize current evidence on the contribution of B-cells and Abs to immunity toward Mtb, their potential utility as biomarkers, and their functional contribution to Mtb control.

## Introduction

Tuberculosis (TB), caused by *Mycobacterium tuberculosis* (Mtb), remains a significant health threat to mankind and is undoubtedly the most successful disease caused by a single infectious agent ever (1). TB killed ~1.5 million individuals in 2018 alone, and a total of around 1,000,000,000 people over the last 200 years (2, 3). In fact, approximately one-fourth to one-third of the world's population is infected with Mtb, giving rise to an estimated 10 million new cases annually (2). Mtb-infection leads to a spectrum of infectious states ranging from various levels of asymptomatic states, collectively referred to as latent tuberculosis infection (LTBI) and to a spectrum of active tuberculosis diseases (ATB), ranging from local to pulmonary to disseminating ATB (4, 5). About 5–10% of individuals with LTBI will progress to ATB during their lifetime; the remainder is able to contain the infection lifelong unless immunosuppressed, such as by coinfecting viruses [e.g., human immunodeficiency virus (HIV)] or iatrogenically (1, 6–8). These data highlight the high level of adaptation of Mtb to infect, and survive in the human host (7).

TB control is hampered by the lack of an effective vaccine: the efficacy of the only available vaccine, *Mycobacterium bovis* Bacillus Calmette-Guérin (BCG), ranges from 0 to 80% (9). A much better understanding of the (protective) immune response to Mtb, the mechanisms by which Mtb manipulates the host response and the identification of robust correlates of protection are all urgently needed to combat this deadly infection.

Large scale, unbiased approaches using advanced -omics technologies analyzing blood samples have been performed over the last decade and identified biomarkers associated with the different disease stages of TB, i.e., which could differentiate LTBI from ATB. In addition, biomarkers for the risk of progression from LTBI toward ATB were uncovered in several large prospective studies (8, 10–15). A frequently appearing transcriptional biomarker which was often a component of signatures able to distinguish ATB from LTBI was *FCGR1A*, a gene encoding the activating high-affinity crystallizable fragment (Fc) gamma receptor I (FcγRI; CD64) (15–22). Fc-Receptors (FcRs) potentially can engage antibodies (Abs) that have opsonized Mtb, and thereby impact mycobacterial survival. Furthermore, in many transcriptomic studies also components of the complement pathway were identified, predominantly transcript-markers from the classical pathway, that were differentially expressed in the blood of ATB compared to LTBI: in particular Complement component1qB (*C1QB)* and *C1QC* were higher expressed, and in support of this, serum C1q-protein was found to be a diagnostic biomarker for ATB (18, 20, 23–25). More recently, it was reported that the combined measurement of serum C1q and whole blood type-1 interferon (IFN) signature might help improving the diagnosis of ATB (26). Together, these studies hint to the potential influence of humoral immune components in TB, including innate and possibly also adaptive humoral immunity. Indeed, in support of this initial data, B-cells and Abs were later proposed to correlate with protective immunity against TB (4, 6, 27–31). This review will explore the role and possible utility of B-cells and Abs as biomarkers of immune protection against Mtb.

**Figure 1:**
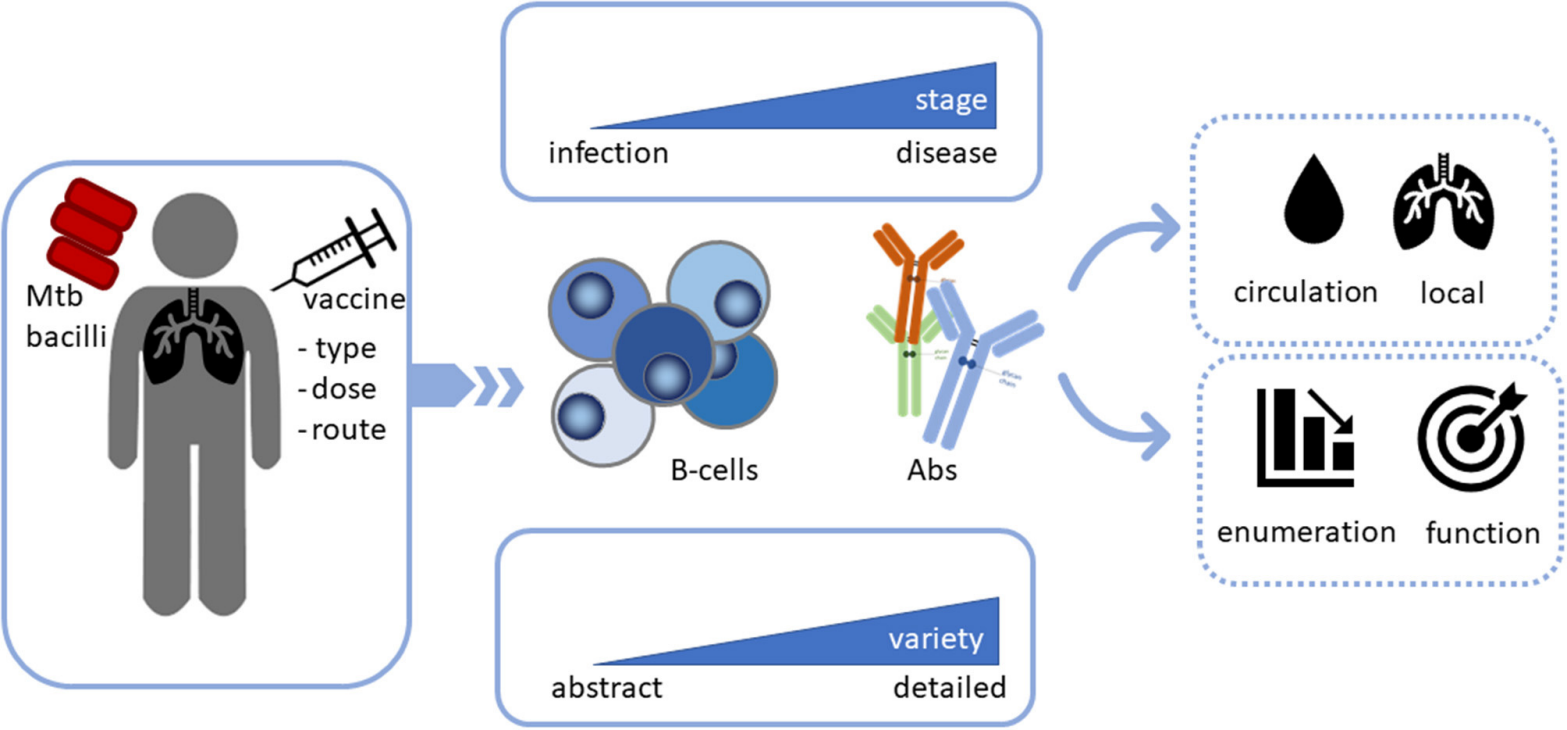
Analysis of B-cells and antibodies (Abs) during Mtb infection, disease and vaccination not only as potential biomarkers, but also as potential functional contributors in the combat against Mtb. Infection with or vaccination against Mtb may activate B-cells as key players, however, different stages of infection may affect B-cell function differentially. The level of detail during assessment is critical as B-cells, as well as antibodies exist in multiple different varieties. The contribution of B-cells and Abs to disease outcome depends on their localization. Finally, it is important to assess their function rather than only enumerate or quantify their frequencies to obtain insights in their Mtb clearing capacity.

As a facultative intracellular bacterium that residues primarily in lung alveolar macrophages, the vast majority of TB research efforts has traditionally focused on understanding cell-mediated immunity (CMI) [reviewed in Cooper (32), Lin and Flynn (33), Ottenhoff (34), North and Jung (35)]. By contrast, the role of B-cell- and antibody-mediated immunity (AMI) in TB has remained understudied for decades. This was due to the historical dogma, established in the early twentieth century, that postulated that host defense against intracellular pathogens is mediated by CMI, whereas the response to extracellular pathogens is mediated by Abs produced from B-cells (4, 7, 36–39). B-cells, however, do not only produce Abs, they are also competent antigen (Ag)-presenting cells (APCs), and produce a wide range of cytokines. All of these B-cell properties can influence the function of a broad range of other immune cells, including T-cells, macrophages, neutrophils and dendritic cells in their response to pathogens (7, 37). AMI combats extracellular pathogens via various mechanisms, such as viral and toxin neutralization (e.g., neutralizing extracellular microorganisms or their products), opsonization (e.g., facilitating bacterial phagocytic uptake by, and recruitment of neutrophils) and complement activation, which can further enhance opsonization and bacterial lysis, but also phagocytosis through complement receptors (40, 41). The effector mechanisms used by specific Abs to remove pathogens is dependent on a variety of features, which include Ag specificity, Ab isotype and subclass, as well as post-translational modifications, like glycosylation (42) (Figure 1).

As Mtb is an intracellular pathogen, Mtb-specific Abs were classically considered to be unable to gain access to phagosomal Mtb bacilli (43). However, several experimental studies in recent decades have shown that B-cells/AMI can contribute to protective immunity, or at least considerably influence host defense, against pathogens with a preferred intracellular niche, such as *Chlamydia trachomatis* (44, 45), Salmonellae (46–48)*, Ehrlichia chaffeensis* (49), *Leishmania major* (50), *Leishmania amazonensis* (51), *Leishmania panamensis* (52), *Cryptococcus neoformans* (53, 54), *Coxiella burnetii* (55), *Trypanosoma cruzi* (56), *Francisella tularensis* (57), and *Plasmodium chabaudi chabaudi* (58). Similarly, proof that B-cells and certain Abs can modulate TB disease is gradually accumulating as discussed in this review and previously elsewhere (7, 29–31, 37, 59–64). The evidence for a contribution of B-cells and antibodies to Mtb clearances varies greatly, which variability may be partly the result of genetic or environmental differences in study populations, which are in particular present in the diverse populations studied in the context of TB, which supports the need of more global assessment of effector responses, including humoral responses. Moreover, although studies in experimental animals have been highly informative, the immune system, including the B-cell compartment, differs amongst species and cannot be completely extrapolated to human infectious diseases. Therefore, a more comprehensive unbiased approach to investigate the functional involvement of cellular immunity, B-cells and humoral immunity, their relative importance, as well as their interconnections in protective immunity against Mtb could enhance our understanding of host defense in Mtb and ultimately might translate into the development of more efficacious therapeutic and preventive tools.

## The Role of B-Cells in Host-Defense Against Mtb

The contribution of B-cells and Abs to immunity against Mtb has been investigated for over 100 years, but results have been inconsistent and sometimes even contradictory (64, 65). A plethora of human and animal studies has suggested though that B-cells and Abs contribute to resolution of TB (Figure 1).

In the past two decades, several studies have reanalyzed the role -protective, neutral or detrimental- of B-cells in TB, using various experimental murine TB-models, including B-cell deficient mice. Some of the results were not in full agreement or even contradictory, with reports showing reduced immunity, delayed dissemination, or even marginal or no detectable effects following genetic deletion (66–73). Both short-term (72) and long-term (70, 71) aerosol infections using virulent Mtb-strain H37Rv (72), Erdman strains (70) or clinical isolate CDC 1551 (71) [with inocula of ~50-100 (70, 71) or 100–1,000 (72) viable bacilli per lung], showed no detectable differences in lung bacterial loads in wild type (WT) vs. B-cell deficient mice. Conversely, studies administering a higher intravenous (73) or intrabronchial (69) dose of Mtb, 10^6^ Mtb H37Rv (73) or four to eight colony forming units of Mtb Erdman (69), respectively, reported an augmented susceptibility to infection, as measured by tissue bacterial burden, in B-cell deficient compared to control mice. Adoptive transfer of B-cells reversed the increased lung immunopathology in B-cell knock-out mice, demonstrating a contribution of B-cells to the control of Mtb (69). In concordance, a high-dose aerosol infection murine TB-model reported exuberated pulmonary pathology with enhanced pulmonal neutrophil recruitment in B-cell deficient mice (67). This study additionally showed that subcutaneous BCG-vaccination elicited an impaired Th1 response in the absence of B-cells. In another vaccination study, adoptive B-cell transfer did not augment anti-TB protection in B-cell knockout mice, but protection required the presence of T-cells (66). However, here the human adenovirus-based vaccine was a powerful T-cell activator that might have acted independently of B-cells.

Extending beyond mouse models, in a CD20^+^ B-cell depleted acute Mtb-infected cynomolgus macaque model, analysis of individual lesions revealed that some, but not all, lesions contained an increased mycobacterial burden and lower levels of inflammation compared to non-depleted animals (74). Thus, despite studies reporting a detrimental or neutral effect of B-cells on anti-TB immunity, there is also increasing evidence for B-cells in promoting optimal protection against Mtb. The contradictory data from studies in B-cell deficient mice may have resulted from differences in the dose and route of delivery, the phase of infection, the mycobacterial strain and the mice strains used (67–72) as was also previously discussed (7).

## The Role of Abs in the Host Defense Against Mtb

### Passive Transfer Studies

A major breakthrough in the immunology and treatment of infectious diseases was the discovery of serum therapy in the mid-1890s (65, 75). A comprehensive account of these early and later studies (from 1880 until mid-1990s) and their limitations was reviewed by Glatman-Freedman and Casadevall (65). Three passive polyclonal immunoglobulin (Ig)G or serum transfer studies provided support for the protective nature of immune serum against Mtb (76–78). Serum therapy with polyclonal Abs against Mtb effectively protected against disease reactivation in Mtb-infected severe combined immunodeficient (SCID) mice after partial treatment with anti-tuberculous drugs (77). In addition, human high-dose intravenous immunoglobulin (IVIg) administration to Mtb-infected C57BL/6, but not nude mice, induced a substantial decline in mycobacterial numbers in the lungs and spleen (78). Sera from some LTBI or highly exposed, but uninfected, healthcare-workers contained protective Abs as shown by serum transfer into mice challenged with aerosol Mtb (76). Thus, in spite of the mixed results obtained in the early passive transfer studies, newer reports underscore the protective capacity of some, but not all, sera and Abs against Mtb. These results call for detailed characterization of the precise properties of polyclonal Ab responses capable of reducing mycobacterial burden.

### Monoclonal Antibody Therapeutic Studies

The development of the hybridoma technology in 1975 provided a tool to overcome the limitations of passive serum transfer with polyclonal Abs, through the production of monoclonal antibodies (mAbs). At the end of the 1990s, one of the first studies that generated mAbs against Mtb evaluated the capacity of three mAbs to influence the course of infection in mice that mainly displayed progressive TB disease (79). Only one mouse mAb, an IgG3 mAb (clone 9d8) specific for the mycobacterial capsular polysaccharide arabinomannan (AM), was able to prolong survival after Mtb challenge via improved tuberculous granulomatous containment of the pathogen (80). Since this pioneering study, several independent studies exploiting mAbs, including different isotypes, IgA (81–88), IgG1 (89), IgG2b (90), and IgG3 (91), against diverse mycobacterial Ags, such as α-crystallin (Acr) (81, 83–88), MPB83 (90), lipoarabinomannan (LAM) (89), and heparin-binding hemagglutinin adhesin (HBHA) (91), have additionally reported protective potential. Efficacy was evaluated by prolonged survival time (89, 90), reduced dissemination (91), diminished tissue pathology (83, 85, 87, 90) and decreased mycobacterial burden as assessed through colony-forming units (82–89). The antibody-isotype is critical for effector function as switching the constant region of the IgA monoclonal 2E9 to Acr abrogated the protective efficacy (81). Interestingly, adoptive transfer of IgA combined with IFN-γ had a strong effect on the bacterial load in a multi-drug resistant Mtb model in mice. Further passive transfer studies of mAbs preferably side-by-side, and including a BCG-immunized control group for referencing, are required to compare and validate the effects of Ab-mediated protection against the tubercle bacillus. Moreover, the development of human or humanized mAbs toward key Mtb epitopes might further define protective humoral responses with significant preventive and/or therapeutic potential.

### Human Observational Studies

In addition to passive transfer and mAb therapeutic studies, insights were also obtained from human observational studies. A meta-analytic study in China showed that patients with X-linked agammaglobulinemia (XLA), a deficiency that results in the absence of B-cells and serum Igs, did not have an elevated risk to developing ATB (92). Likewise, patients with common variable immunodeficiency (CVID) did not have increased susceptibility to TB (93). However, these observations could be confounded by IVIg therapy given to most patients (92, 93). Moreover, an argument often used to argue against a significant role of Abs in the control of Mtb is that humans treated with Rituximab, a B-cell depleting anti-CD20 mAb, did not have an increased risk of reactivating TB (94, 95). Counter arguments, however, include that Rituximab has a limited depleting effect on CD20-negative Ab secreting plasma cells and is also not able to modify pre-existing Ab levels, thus not excluding that the absence of increased susceptibility seen in these patients might be the result of remaining Abs (94, 95). Moreover, Rituximab is mostly used in developed countries where TB-incidence and thus the risk of acquiring Mtb-infection is very low. In addition, patients about to start this treatment are routinely screened for LTBI and will first receive preventive antibiotic treatment before initiating anti-CD20 therapy. Hence, the argumentation that Rituximab does not induce an elevated risk on acquiring TB is not convincing.

While the first two studies described above (92, 93) provide an observational analysis in populations characterized by the absence of Abs, other studies have investigated the potential protective roles for Abs in Mtb containment more directly. Costello et al. reported that children from the United Kingdom and Southeast Asia with disseminated ATB disease had lower LAM-specific IgG serum-titers in comparison to individuals with localized ATB lesions (96). In agreement, the decrease in LAM IgG-titers from placentally transferred maternal IgGs until increased production of infant IgGs correlated with the peak incidence of disseminated ATB (96–98). Similarly, the absence of Abs binding to the mycobacterial 38 kDa Ag correlated with disseminated TB in children and TB-meningitis in adults (99). In addition, other serological studies have also found lower Ab serum-titers in both children and adults with extrapulmonary, active and/or disseminated TB (100–105). These human observational findings implicate that some Abs specific for particular Ags could contribute to Mtb control although causality cannot be established.

## The Mechanistic Role of B-Cells in TB

Intriguingly, B-cells and Abs are not only detected in the circulation, but are also hallmarks of TB-associated granulomas, the highly organized structures formed in the lung to contain the bacilli. In particular, B-cells in the tuberculous lung have the ability to form aggregates that display features of germinal centers (GCs), bona fide organizational marks of secondary lymphoid tissues.

### B-Cell Organization in Ectopic Germinal Center-Like Structures in Tuberculous Granuloma in the Lung

#### Structural Organization of Granulomas in Tuberculous Lungs

B-cells are components of the granulomatous lesions in the lungs of Mtb-infected mice (69, 106–111), non-human primates (NHPs) (112), and ATB patients (106, 110, 113, 114). A classical TB-granuloma contains a central region, which usually comprises Mtb-infected macrophages and can be infiltrated with neutrophils; can develop into necrotic with caseous cellular debris, or alternatively form mineralized lesions (7). Surrounding this necrotic center is a layer of foamy and epithelioid macrophages interspersed with Langhans giant-cells, which in turn is surrounded by an outer layer of lymphocytes scattered with macrophages (115, 116). At the periphery, B-cells form highly organized structures resembling B-cell follicles of secondary lymphoid organs, which are called tertiary lymphoid organs, ectopic lymphoid follicles or, if formed in the lung, inducible bronchus-associated lymphoid tissue (iBALT) (61, 117). These lesional B-cell aggregates are the predominant site of immune proliferation in the lungs of pulmonary ATB patients (114). Furthermore, immunohistochemical and flow cytometric characterization demonstrated the presence of peanut agglutinin and GL7 (two GC markers) expressing B-cells, CD68^+^ macrophages, central CD21^+^ follicular dendritic cells, CXCR5 (CXC chemokine receptor 5)^+^ inducible co-stimulator^+^ CD3^+^ T-cells (e.g., classical T follicular helper cell) and tissue expression of CXCL13 (CXC-chemokine-ligand-13) in these TB-ectopic aggregates (69, 106). Together, this indicates that B-cell follicles (BCFs) in the proximity of TB-granulomas are ectopic GCs at the cellular, molecular and structural level (117, 118) and are therefore, presumably, the product of lymphoid neogenesis, a highly complex process that occurs during chronic inflammation where GC-like structures are formed ectopically in non-lymphoid tissues [reviewed in Pitzalis et al. (118), Aloisi and Pujol-Borrell (119)].

#### Role of Ectopic B-Cell Follicles in Granulomas During Mtb-Infection

The impact of ectopic BCFs on the course of Mtb-infection is unclear (115, 117). Kondratieva et al. reported that an abolished lung granulomatous architecture had no effect on severity of Mtb-infection in mice (120). Likewise, Slight et al. claimed that BCF-formation does not control tubercle bacillary growth, but that formation of these follicles is merely a result of correct CXCR5^+^CD4^+^ T-cell localization within the lung-parenchyma (106). On the other hand, Maglione et al. have shown that B-cell deficient mice, lacking BCFs in the lungs, have abnormal granulomatous responses correlating with augmented pulmonary pathology and suboptimal bacterial control (69). Moreover, during Mtb-infection CXCR5^+^CD4^+^ T-cells accumulated within BCFs and locally produced proinflammatory cytokines required for effective macrophage activation and optimal bacterial control (106). Yet, in disordered BCFs associated with irregular CXCR5^+^CD4^+^ T-cell localization there was no protection against Mtb indicating a protective rather than deleterious role of organized BCFs (106, 110). In agreement, lung BCF formation in ATB-patients correlated with containment of lung Mtb-infection (121). In a murine TB-model, the presence of ectopic B-cell aggregates was associated with granuloma formation and prevention of Mtb dissemination (108). Mtb and host-specific triggers that engender protective vs. pathological outcomes for BCFs are just starting to be elucidated, but probably include Ag type, type and duration of the response induced, as well as the type of Ig-subclasses involved (117, 122). Likely Mtb factors are directly guiding the organization of the granulomas and their associated BCFs (115). Collectively, this suggests a potentially protective role for ectopic pulmonary B-cell follicles during Mtb-infection.

### B-Cell Phenotypes and Frequencies During Mtb-Infection

**Figure 2:**
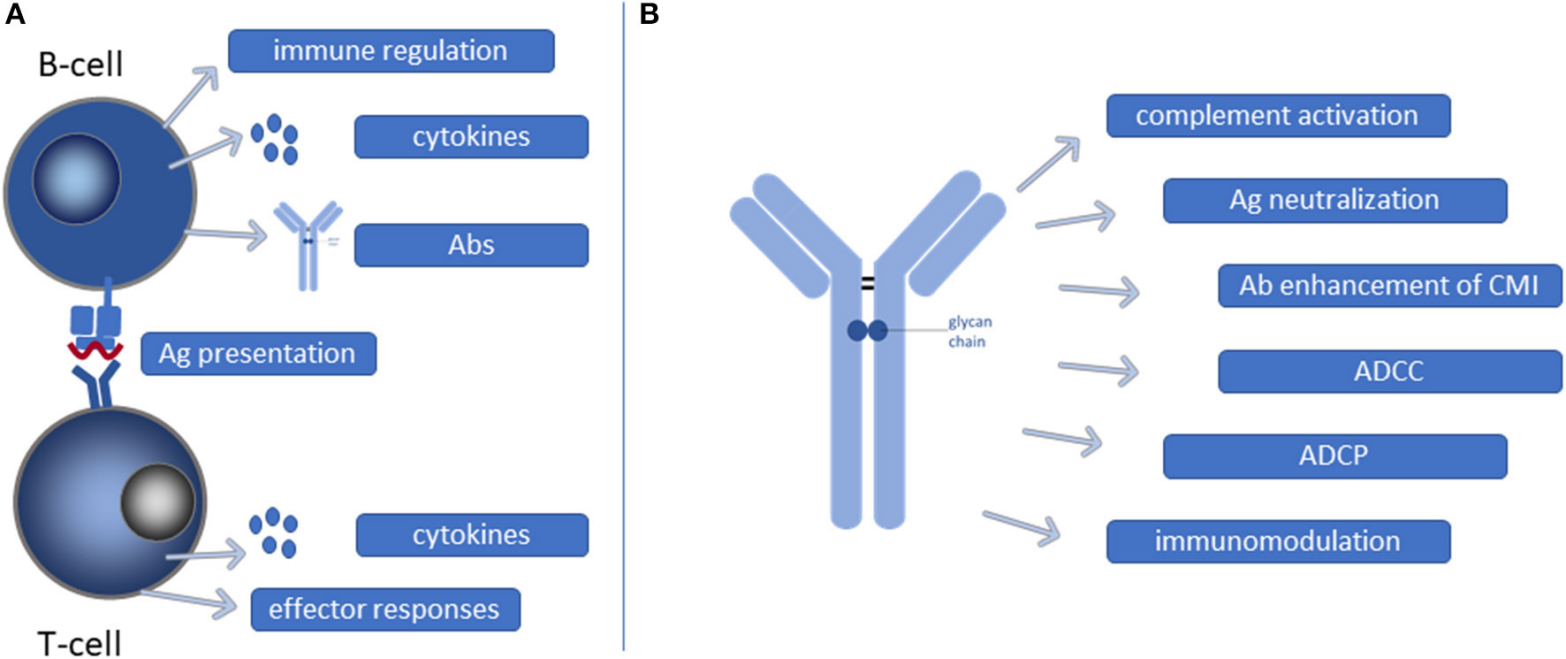
Functional contribution of B-cells and Abs during Mtb infection. **(A)** B-cells may present mycobacterial antigens (Ags) as professional antigen presenting cells to T-cells resulting in T-cell activation and CMI. Moreover, B-cells, as well as activated T-cells, secrete cytokines that contribute to shaping of effector responses. Furthermore, B-cells can also have direct immunoregulatory functions and secrete Abs. **(B)** Ab binding to Mtb can initiate a multitude of different processes, each contributing in their own way to reduction of the mycobacterial burden. CMI, cell mediated immunity; ADCC, antibody dependent cellular cytotoxicity; ADCP, antibody dependent cellular phagocytosis.

The functional role of B-cells has mostly been investigated by assessing total B-cells, however, B-cells exist in multiple flavors, each with unique properties and contributions to the host immune response. Detailed assessment of these different roles may provide an additional level of depth in the understanding of B-cell function during TB (Figure 2A).

Enumeration of B-cells during clinical TB has yielded contradictory results (116, 123–127). Significantly decreased B-cell frequencies were detected in the peripheral blood of ATB (116, 123–125) or LTBI (124) individuals compared to healthy controls. Others reported unaltered (124, 127), or even increased (126) B-cell frequencies in the blood of ATB. These conflicting data are likely the result from differences between study designs and groups of patients enrolled, varying by age, gender, ethnicity, form and severity of TB disease (61).

#### B-Cell Subsets During Mtb-Infection

Only few studies have assessed B-cell subset frequencies and relative changes during Mtb-infection (60, 116, 128–133). Patients with ATB and LTBI had, in comparison to healthy controls, elevated circulating populations of atypical (CD21^−^CD27^−^ or IgD^−^CD27^−^) and activated (IgD^−^CD27^+^) B-cells, whilst the population of naïve (IgD^+^CD27^−^) B-cells was reduced in both patient groups (116). Atypical B-cells were not only increased in frequency, but where also functionally impaired with reduced proliferation, cytokine and Ab production. Upon successful antibiotic treatment, B-cell numbers and function were restored (116, 134). These frequency differences were confirmed in another study involving patients with ATB, other lung diseases, and following ATB-treatment (129). The circulating proportion of non-class switched marginal zone (CD19^+^IgM^+^CD27^+^CD23^−^) and class-switched mature (CD19^+^IgM^−^) B-cells was significantly lower in ATB-patients compared to other lung diseases, suggesting that Mtb-infection suppresses and/or exhausts B-cell effector functions, comparable to what has been observed in HIV-positive people (135). In contrast, memory B-cells (CD19^+^IgM^−/+^CD27^++^), plasmablasts (CD19^+^IgM^−/+^CD138^+^CD27^+^), memory-plasmablasts (CD19^+^IgM^−^CD138^+^CD27^++^) and circulating marginal zone (CD19^+^CD27^+^CD23^−^) B-cells were significantly increased at diagnosis compared to post-treatment, suggesting their potential utility as TB-treatment response biomarkers (129). A study comparing multi-drug resistant-TB patients with healthy controls revealed decreased rates of non-class switched memory (IgD^+^CD27^+^) B-cells and circulating plasma cells (CD19^dim^IgD^−^CD38^+++^CD27^++^), with increased numbers of circulating type-1 transitional (IgD^+^CD38^++^), CD69^+^ and Toll-like receptor 9 (TLR9)^+^ B-cells in the peripheral blood of multi-drug resistant-TB patients (131). Another study reported increased frequencies of marginal zone (CD19^+^CD21^+^CD23^−^) B-cells in QuantiFERON-TB Gold In-Tube test (QFN) positive compared to QFN-negative individuals (136).

Studies characterizing and enumerating B-cell phenotypes during TB not only analyzed the presence of various B-cell subpopulations in peripheral blood, but also in the lungs and pleural cavity. Approximately 85% of B-cells present in both unaffected and Mtb-infected mouse lungs expressed surface markers typical of follicular B-cells or B2-cells (e.g. CD19^+^B220^high(hi)^IgM^low(lo)^IgD^hi^ with the phenotype CD21/35^+^CD11b^−^CD1d^−^CD5^−^CD43^−^), whilst 15% exhibited a surface expression that is characteristic of antibody producing B1-cells (e.g. CD19^+^B220^lo^IgM^hi^IgD^lo^CD23^−^CD5^−^/CD5^−^) (128). In the lungs, the B1/B2 ratio was comparable between infected and uninfected mice. In the pleural cavity, however, progression of TB was associated with an elevated proportion of the B2-population (from 25 to 60%) (128).

Another important B-cell subset are regulatory B-cells (Bregs), which balance immune activation following inflammatory responses during infection. An increased frequency of CD19^+^CD1d^+^CD5^+^ Bregs in the circulation of ATB-patients coincided with increased inhibition of T-helper (Th) 17 responses and interleukin-22 (IL-22) generation, while Th1 responses remained unchanged (132, 137). The fact that this Breg-subpopulation was also present in healthy donors, but with decreased suppressive activity, suggests that CD19^+^CD1d^+^CD5^+^ Bregs have activity regulated in response to infection (137). Furthermore, patients with cavitary TB, a severe clinical manifestation of ATB, had increased numbers of CD19^+^CD1d^+^CD5^+^ Bregs in the peripheral blood in comparison to ATB-patients without cavitation (132). Thus, CD19^+^CD1d^+^CD5^+^ Bregs might dampen protective anti-Mtb effector responses, and indeed Mtb Ag-specific IL-22 responses during ATB-treatment were related to reduced CD19^+^CD1d^+^CD5^+^ Breg numbers (132).

Another type of Bregs includes a rare subset, called killer B-cells (CD19^+^CD5^+^IgM^+^), which are characterized by Fas-ligand (FASL, CD178) expression (138). Reduced levels of *FASL* transcript, a diminished incidence of FASL expressing B-cells and a lower level of soluble FASL were detected in the bronchoalveolar lavage (BAL) fluid of ATB-patients at diagnosis compared to the end of successful anti-TB treatment (139). The expression of *FASLG* and *IL5RA* was lower in ATB-patients compared to healthy controls, but upon anti-TB treatment, levels were completely restored (140). The frequency of FASL expressing B-cells was lower in whole blood from ATB-patients compared to healthy controls (138). FASL expressing B-cells were present during both ATB and LTBI, but the frequency of this Breg-subset was higher in LTBI and was even further elevated after B-cell re-stimulation with BCG (138). Thus, killer B-cells may contribute to protective immunity during Mtb-infection. Further work is required to decipher the exact role of killer B-cells during Mtb-infection.

Overall, these data implicate that the relative frequencies and function of B-cell subsets are affected during Mtb-infection, “protective” B-cell subsets are decreased in numbers, whilst potentially pathological B-cell subpopulations are increased during ATB-disease. Importantly, patients cured from ATB-disease have normalized B-cell numbers, with normal phenotype distributions and functional properties, indicating restoration of the responses.

### B-Cell Modulation of Effector Cells During Mtb-Infection

B-cells are players in the formation of effective immune responses since B-cells are not only potent APCs, but also represent powerful producers of a wide range of cytokines and Abs (7, 37). All of these B-cell features can influence the function of a broad range of immune cells, such as T-cells, macrophages, neutrophils and dendritic cells.

#### B-Cells Guiding T-Cell Responses

##### B-Cell Antigen-Presenting Capacity as Modulator of T-Cell Immunity

The ability of B-cells to function as APCs may contribute to the orchestration of Mtb specific CD4^+^ T-cell immunity (60). Surprisingly, Ag presentation by B-cells during Mtb-infection has hardly been assessed (128, 141). In B-cell deficient mice, it was demonstrated that correct programming and induction of effector cells specific for Mtb Ags necessitated presentation of these particular Ags to CD4^+^ T-cells by B-cells (141). Moreover, aerosol challenge of genetically Mtb-susceptible I/St mice slightly increased the level of major histocompatibility complex-class II molecules on the surface of lung B-cells during Mtb-infection, and their efficacy to present Mtb Ags to CD4^+^ T-cells was comparable to that of their splenic counterparts (128). Importantly, the APC-function of B-cells during Mtb-infection appears to become progressively more relevant at lower Ag load, but likely superfluous at higher Ag loads (142, 143). The Ag-presenting potential of B-cells was also shown to increase vaccine-effectiveness (including TB-vaccines), and to strongly boost BCG primed immunity (144–146).

##### B-Cells and Their Cytokines as Powerful T-Cell Rheostats

B-cells isolated from lungs of Mtb-infected mice produced and released a wide range of cytokines (7, 37). In a cynomolgus macaque (*Macaca fascicularis*) model a role for granulomatous B-cells in producing IL-6 and IL-17 was discovered, and to a lower degree IL-10 and IFN-γ, during the acute phase of Mtb-infection (74). B-cell depletion, however, only resulted in diminished secretion of IL-6, but not IL-17 (74), suggesting functional cellular interactions for B-cells in lesions. In agreement with the ability of B-cells to produce IL-6, Linge et al. demonstrated that lung B-cells in I/St mice can secrete high to moderate levels of proinflammatory IL-6 and IL-11 when challenged with Mtb-strain H37Rv (128). Atypical B-cells with reduced production of intracellular IL-6 were isolated from the blood of both ATB and LTBI patients (116). Taken the diverse stages of TB disease into consideration, du Plessis et al. attempted to map the B-cell derived cytokine profile during LTBI (147). B-cells could produce both pro- and anti-inflammatory cytokines when stimulated with Mtb (or TLR agonists), including IL-1β, IL-10, IL-17, IL-21 and tumor necrosis factor-alpha (TNF-α) (147).

B-cells produce and release specific cytokines upon activation, and thereby a.o. shape (T-cell) immune responses against Mtb. Specifically, IL-1β and IL-6 have been reported to play a critical role in establishing and sustaining Th17-responses to Mtb (148–150). In addition, IL-6 directs differentiation of Th1-cells from naïve T-cells (151). Although the function of IL-11 in anti-Mtb immunity remains to be elucidated, in multiple sclerosis this cytokine is a potent stimulator of Th17 responses (152, 153). Moreover, IL-21 has been shown to play a critical role in T-cell immune responses against Mtb by enhancing CD8^+^ T-cell priming, increasing T-cell accumulation in the lungs and potentially by inhibiting T-cell exhaustion (148). Similarly, TNF-α has been shown to enhance T-cell responses through augmenting Ag-presentation and cross-priming (154). Furthermore, both TNF-α and IL-17 can regulate chemokine-expression and thereby modulate the recruitment and maintenance of immune cells, including T-cells, at the site of infection (155–157). In contrast, IL-10 can dampen Mtb-specific Th1 responses through inhibition of TNF-α and the Th1-polarizing cytokine IL-12 and human leukocyte antigen-class II expression, thereby limiting Ag-presentation, cross-priming and migration of Th1-cells toward the lungs (158–161). Collectively, these data point to an important role for B-cell produced cytokines in the generation and regulation of CMI to TB.

## The Mechanistic Role of Abs in TB

Besides their antigen presentation capacity and their ability to skew T-cell responses through cytokine secretion, B-cells are best known for their capacity to secrete Abs. Like B-cells, Abs exist in multiple isotypes and subclasses, each with distinctive functional properties, which are further diversified by post-translational modifications, representing an enormous functional potential for Abs in effector immunity toward Mtb (Figure 2B). TB disease, but not Mtb infection itself, may significantly alter the functional properties, including the avidity, of Abs against heterologous (162), reflecting modification of humoral responses by Mtb likely by altering B-cell function. In this chapter, we will focus on the Mtb specific responses.

### Abs as Potential Biomarkers for Protective Immunity Against Mtb

“Natural immunity” against Mtb has recently been studied in various human “resistor” or “early clearers” cohorts, amongst which were health care workers (76), household TB contacts (163–165) and gold miners (166). These studies consistently identified that ~5–15% of the tested individuals in a TB-endemic region are resistant to acquire latent Mtb-infection as determined by tuberculin skin test (TST) or QuantiferonTB Gold (QFT) conversion (167). Genome-wide linkage analysis in a panel of South African families living in a hyperendemic area demonstrated that the locus called *TST1* was associated with TST reactivity (168). A deep sequencing study showed preferential rearrangement of V_H_3-23-D3-3-J_H_4 fragments in IgA molecules in TST-negative nurses with long-term exposure to Mtb compared to their TST-positive colleagues (169). Moreover, healthy nurses in a TB ward had a strong Ab-response specific toward the TB69 epitope of the 14-kDa Ag, possibly linked to resistance to acquiring Mtb-infection (170). Individuals with persistent negative TSTs, despite years of exposure to ATB patients, had elevated anti-Mtb IgG levels, and their serum was able to block proliferation of peripheral blood mononuclear cells in response to protein purified derivative (PPD) (171). In concordance, highly exposed, but TST-negative, Colombian individuals displayed high anti-PPD Abs titers, which inhibited autologous T-cell proliferation after PPD stimulation (172). Abs specific for CFP-10 and ESAT-6 in QFT supernatants independently separated LTBI from ATB (173). More recently, Lu et al. reported that highly exposed, but TST- and IFN-γ release assay (IGRA)-negative, Ugandan individuals harbored Mtb-specific IgM and IgG, while diminished CD4-mediated IFN-γ responses directed toward Mtb early secreted Ag of 6 kDa (ESAT-6), 10 kDa culture filtrate protein (CFP-10), Ag85A and Ag85B were found (163). Taken together, these studies implicate that humoral immunity is detectable in frequently exposed individuals with persistently negative skin testing or QFN evaluation, which represent read-outs of effector T-cell responses. In such settings, Abs may be considered biomarkers of protective immunity.

### Ab Effector Functions Against Mtb

Although Abs may be interesting biomarkers of Mtb-infection or resistance to disease progression, they may also contribute functionally to reduce bacterial loads. However, when considering a role in prevention of infection Abs need to localize to sites where the pathogen enters the host to inhibit, or contribute to early clearance of, infection. In addition, Abs need to trigger the right effector responses, therefore functional assessment rather than mere quantification is critical to evaluate the contribution of Abs to the immune response. Typically, Abs are located in both the upper and lower respiratory tract, where IgA dominates in the upper airways and IgG in the lower airways. It has been shown that human infection with Mtb resulted in mycobacterial specific IgA and IgG in BAL fluid (174, 175). Abs can bind Mtb-specific Ags at the site of disease (30, 60), such as the tuberculous granuloma where plasma cells have been demonstrated to secrete Abs (112), which could potentially interact with extracellular Mtb and/or free Mtb Ags present in the granuloma itself, or in the pleural fluid (112, 176, 177). In addition, Mtb is extracellular during its reinfection phase and during expectoration. Thus, in spite of being a facultative intracellular pathogen, Mtb is possibly susceptible to numerous mechanisms of AMI (6, 178). These comprise, but are not limited to, mycobacterial neutralization (82, 91, 179), antibody-dependent cellular phagocytosis (ADCP) (180, 181), complement activation (182–184), antibody-dependent cell-mediated cytotoxicity (ADCC) (185), Mtb-Ab immune complex sensing by intracellular FcR tripartite motif-containing protein 21 (TRIM21) (186, 187), stimulation of CMI (43, 188, 189) and modulation of the strength and nature of the inflammatory response during Mtb-infection (6, 31, 36, 69, 185, 190–193).

#### Ab-Dependent Opsono-Phagocytosis of Mtb

One of the most important Ab effector functions against Mtb is opsono-phagocytosis, also called ADCP (63). ADCP is mediated by mononuclear phagocytes and granulocytes upon engaging FcRs or, following complement opsonization, complement receptors (42). Mtb inhibits phagosome-lysosome fusion to evade exposure to the antimicrobial lysosomal content (194–197). However, Ab-mediated phagocytosis of opsonized mycobacteria can overcome this inhibition by triggering phagolysosomal fusion (197). Similarly, more recent studies have found increased phagosome maturation in the presence of opsonizing Abs and showed decreased mycobacterial viability upon phagolysosomal fusion (180, 181, 189, 198). Opsonizing Abs restricted Mtb growth in macrophages by significantly increasing the microbicidal potency through increased lysosomal-associated membrane protein 1 (LAMP-1; a phagosome maturation marker) and inducible nitric oxide synthase (iNOS) phagosomal localization and enhanced phagosome acidification, as well as by increased levels of the proinflammatory cytokines IFN-γ and IL-6 (194). Another study described that IgG-coated BCG induced increased microbicidal activity by alveolar macrophages associated with an elevated oxidative burst in phagosomes (199). Mtb opsonized with LAM-specific Abs bound FcγRs on macrophages and stimulated mycobacterial killing, which was associated with increased calcium (Ca^2+^) concentrations that promoted phagosome maturation (196). Uptake of Mtb mannose-capped LAM (ManLAM) beads into human alveolar macrophages via mannose receptors initiated a specific phagocytic pathway that limited phagosomal fusion, whereas phagocytosis of anti-LAM mAb coated beads through FcγRs did not inhibit the fusogenic property of phagosomes (200). In agreement with these results, uptake of phosphatidyl-myo-inositol mannoside (PIMs)-coated beads into macrophages via engagement of mannose receptors resulted in restricted phagolysosomal fusion (201). Thus, Mtb cell entry via mannose-, complement- and probably other phagocytic receptors allows intracellular survival and replication of bacilli by limiting phagosome maturation while on the other hand, increased phagolysosomal fusion is observed after phagocytosis of Ab-opsonized mycobacteria via FcR signaling (198). Indeed, Chen et al. have demonstrated that increased Mtb phagocytosis and the subsequent increased phagolysosomal fusion observed in THP-1 cells infected with Mtb opsonized with sera containing high anti-AM IgG titers derived from asymptomatic volunteers, was FcγR mediated (180). Furthermore, a significant decline in Mtb phagocytosis by human anti-Mtb Abs was observed when blocking FcγRI (CD64), which was even more prominent when FcγRII (CD32) was blocked. In contrast to (180), Lu et al. found that enhanced functionality of polyclonal LTBI IgG was correlated with selective FcγRIII (CD16) binding, which was associated with increased phagosome maturation and elevated macrophage killing of intracellular tubercle bacilli (185). Intriguingly, CD16 positive, non-classical monocytes were strongly associated with reduced mycobacterial outgrowth upon recent Mtb exposure (202). The discrepancies between studies can likely be attributed to differences in Abs related to the stage of TB disease, and differences in assays and phagocytosing cells (203, 204), as THP-1 cells do not express FcγRIII (CD16), whilst monocyte-derived macrophages do (205, 206). In summary, Ab opsonization of Mtb and subsequent FcR signaling can target Mtb to the degradative lysosomal pathway, which is directly antimicrobial even though it may not be capable to induce complete elimination (194).

#### Ab-Mediated Classical Complement Activation in TB Disease

An additional mechanism by which AMI could influence the host response against Mtb is through Ab-mediated complement engagement and deposition (182–184). As discussed previously, transcripts of different complement genes were strongly increased during active TB-disease, which were considered important biomarker candidates (18, 20, 23–26). In addition, increased concentrations of circulating immune complexes were detected in serum of patients with subclinical and clinical ATB (24), however, the antigen in these complexes remains unknown. In Indian TB patients anti-LAM IgG2, but not IgM, was associated with classical pathway complement activation (184). Likewise, human IgG, and to a lesser degree IgM, was shown to increase complement deposition on BCG via classical pathway activation (183). Moreover, human anti-Mtb IgG augmented complement activation resulting in enhanced phagocytosis of Mtb by macrophages (182). Thus, complement activation by anti-Mtb Abs seems possible, but has been investigated to a very limited extent, nonetheless, it may significantly contribute to Mtb phagocytosis.

#### Ab-Mediated Cellular Cytotoxicity in TB Disease

ADCC might represent another classical mechanism that could possibly help controlling Mtb. IgG-mediated ADCC might stimulate killing of Mtb and could play a vital role in the early containment of Mtb upon their re-entry into the extracellular space. Indeed, PPD specific IgG from both LTBI and ATB-patients increased natural killer (NK) cell-mediated ADCC (185). IgG derived from LTBI patients revealed a preferential interaction with the activating FcγRIIIa (CD16a) associated with an increased on-rate in comparison to IgG isolated from ATB patients (185). This enhanced FcγRIIIa (CD16a) binding profile correlated with increased NK cell activation, elevated ADCC and enhanced Mtb control. These findings suggest a protective role for ADCC in host defense against Mtb.

#### Intracellular Sensing of Mtb-Ab Immune Complexes by TRIM21

Going beyond Ag-Ab immune complex recognition by classical FcRs, Ab binding to Mtb might also be detected intracellularly via the ubiquitously expressed cytosolic Ab FcR called TRIM21. Interestingly, the TRIM21 pathway was identified as important pathway in TB when interconnectivity of multiple biomarkers was analyzed in unbiased transcriptomic studies, suggesting TRIM21 may not only be a biomarker of TB-disease, but also functionally involved in reducing the bacterial load (8). Ab-coated pathogen binding to TRIM21 has been demonstrated to result in the activation of signaling pathways, including nuclear factor kappa-light-chain-enhancer of activated B-cells (NF-κB), activator protein (AP)-1 and the IFN-regulatory factor (IRF) family, and stimulate the production of proinflammatory cytokines via K63-linked ubiquitination (186, 187). Hence, TRIM21 triggering elicits an anti-pathogenic state and can provide protective immunity against non-enveloped viruses and intracellular bacteria, such as adenoviruses, *Salmonella enterica* and *Toxoplasma gondii* (186, 187, 207, 208). Importantly, a requirement for TRIM21-mediated signaling and neutralization includes relocation of Abs from the extracellular space toward the cytosol, where Abs are normally not found (186, 187). In the case of Mtb, the bacillus is able to disrupt the phagosomal membrane, which allows Mtb together with bound Abs to enter the cytosol (209). Thus, the cytosolic localization of both Mtb specific Abs and TRIM21 in combination with the circumstantial evidence described above might open up attractive new roles for Abs in the battle against Mtb.

### The Role of Abs in Mucosal Immunity Against TB

The cumulative work presented above clearly indicates the pleiotropic effect of Abs in the immune response to Mtb, in which the particular Ab effector function utilized is dependent on Ag specificity, Ab isotype and subclass (28, 42, 59, 210). The pulmonary compartment represents the predominant route of Mtb-infection, and the distribution of Ig isotypes in the mucosal lining includes predominantly secretory IgA (sIgA), lower amounts of soluble IgM, and even lower soluble IgG. Belay et al. have demonstrated that exposed healthy controls have a significantly increased anti-HBHA IgA titer in comparison to untreated TB-patients and their QFN-negative household contacts at baseline, suggesting that anti-HBHA IgA could function as a biomarker for immune control of Mtb (211). Mucosal BCG-vaccination by bronchial instillation in rhesus macaques induced pulmonary IgA (measured in BAL fluid), which correlated with protection against low-dose TB-challenge (212). Interestingly, another strongly protective regimen, intravenous BCG-vaccination, also induced increased levels of pulmonary IgA in NHPs (213). Thus, local IgA may represent a correlate of protection, and may functionally contribute to control of mycobacteria.

For functional assessment, polyclonal human sIgA purified from colostrum from healthy volunteers was found to be reactive to both BCG and Mtb Ags (214). Prophylactic intratracheal incubation or pre-incubation of tubercle bacilli with human sIgA resulted in reduced Mtb viability, which was associated with reduced lung tissue damage, as indicated by better granuloma organization and smaller pneumonic areas in the lungs of Mtb-infected mice. Likewise, when IgA deficient (IgA^−/−^) and WT littermate mice were intranasally inoculated with the mycobacterium surface phosphate-binding protein PstS-1 (215), IgA^−/−^ mice were still capable of generating PstS-1 specific IgM and IgG, but revealed an increased susceptibility to BCG infection in comparison to WT mice: this was indicated by elevated mycobacterial loads in the lungs at 4 weeks post-infection, and decreased production of IFN-γ and TNF-α. Similarly, mice lacking the polymeric immunoglobulin receptor (pIgR) that enables IgA transcytosis, inoculated with PstS-1 Ags, developed decreased PstS-1 specific IgA titers in their saliva (216). These mice additionally showed increased susceptibility to intranasal BCG, which again correlated with an impaired Th1-response, as manifested by diminished IFN-γ and TNF-α levels in the lungs, and with delayed and reduced mycobacterial-induced immune responses during early infection. These studies collectively indicate that sIgA participates in protection against Mtb-infection and this effect is mediated via modulation of the inflammatory response at the mucosal level and/or by immune exclusion, a process that refers to the capability of sIgA to prevent pathogens and Ags from acquiring entry to the epithelium (217, 218).

Given the importance of mucosal IgA in host defense against Mtb (219), significant prophylactic and therapeutic improvement could be achieved when eliciting a mucosal immune response at the port of Mtb entry in addition to a systematic immune response. Passive immunization with mAbs has opened avenues for potential protective roles of mucosal IgA and the ability to explore different routes of delivery and their subsequent effects. In fact, in a mouse model of mycobacterial lung infection, intranasal immunization of mice with an IgA mAb (TBA61) directed against Mtb Acr1, induced a transient reduction in bacterial counts in the lungs following aerosol or intranasal challenge (88). This protective effect was improved and prolonged (3-4 weeks) when the Ab was co-administered with mouse recombinant IFN-γ alone (87) or in combination with IL-4 neutralization (86) and was demonstrated to be both isotype and epitope specific (88). This finding is in agreement with the observation that intratracheal mAb TBA61 pre-treatment resulted in decreased mycobacterial loads in mouse lungs, which was correlated with milder histopathology following Mtb-challenge (85). More recently, it has been shown that in human FcαR (CD89) transgenic mice, intranasal administration of a human IgA1 mAb specific for Acr1 combined with recombinant mouse IFN-γ significantly reduced bacterial load after intranasal Mtb-infection. Thus, the observed protective effect of IgA in this setting was FcαR (CD89) dependent (81, 83). Collectively, IgA mAb can influence the intracellular fate of Mtb via potentiation of mycobactericidal functions of infected macrophages and by modulation of the inflammatory response (87, 219–221).

Together, the work described above highlights the importance of mucosal immunity in host defense against Mtb. Although local IgA production correlated with protection induced by intravenous or mucosal BCG vaccination, its functional capacity to control mycobacteria remains to be demonstrated.

### Glycosylation Patterns as Modulators of Ab Functionality in TB

Modification of Abs, like glycosylation, influences the function of Abs (222). These glycans modify the Ab Fc region structure, and addition or removal of glycan-molecules modifies Ab FcR engagement and Ab functionality (222, 223). Specifically, recent new data obtained from several viral and bacterial diseases implicate that infection, and the associated inflammatory state of the individual, alters Ab glycosylation and hence functionality (224, 225).

Given the dynamic nature of glycan alterations that is impacted by the inflammatory milieu (226), Ab glycosylation changes could also occur during the course of TB disease. Using systems serology, Lu et al. showed that the LTBI Ab glycome of total and PPD-specific IgG had accumulated digalactosylated (G2) glycans with an increased level of sialylation, but less core fucosylation in comparison to ATB IgG (185). This finding resonates with the observation that individuals with ATB displayed a marked elevation of agalactosylated (G0) and asialylated IgG glycans (227–229). Although the absence of galactose is considered to be correlated with increased inflammatory activity in general (223, 226), agalactosylated (G0) IgG could trigger the lectin complement pathway (230). The presence of sialic acid has been correlated with an anti-inflammatory state in rheumatoid arthritis patients (231), and this could reflect diminished inflammation in LTBI compared to ATB patients. Increased FcγRIIIa (CD16a) engagement by polyclonal IgG from LTBI individuals led to increased Mtb-specific ADCC and killing of tubercle bacilli in infected primary human monocyte-derived macrophages in comparison to purified IgG derived from ATB-patients (185). Interestingly, NK-cell frequencies and CD16 function were associated with LTBI (124). Together, these data highlight that the discrete Ab glycosylation patterns observed in LTBI persons might correlate with increased Mtb control.

Removal of polysaccharides from purified IgG decreased the level of ADCP, showing that Ab glycosylation is essential for anti-Mtb activity (185). Glycosylation profiles differed for divergent Mtb reactive Ab populations (e.g., recognizing PPD and Ag85A), suggesting possible differential modulation of glycosylation across IgGs against different Mtb-specific Ags (232). Furthermore, parallel profiling of whole IgG and Ag-binding fragment (Fab) and Fc region-specific IgG glycosylation, showed that the main alterations in Ab glycan moieties across divergent TB-disease states (185) were located in the Fc-region. Stringent multivariate analysis further demonstrated that Fc-region glycosylation can distinguish between LTBI and ATB disease states (232). Digalactosylated (G2) glycan structures and the discrete structure G1S1F (characterized by one galactose, one sialic acid, and one fucose) located on the IgG-Fc region discriminated LTBI from ATB individuals, and likewise distinguished successfully treated ATB individuals from ATB patients (Grace et al., under review). In addition, Mtb-specific IgG4 titers were identified as a novel biomarker for TB disease, in which IgG4 levels were increased during ATB compared to LTBI and individuals that had successfully completed TB-treatment. IgG4-depletion increased Ab effector function, suggesting regulation of the overall humoral response. In addition, LTBI individuals could be distinguished from successfully treated ATB patients by lower Mtb-specific IgM and IgG1 levels and decreased opsono-phagocytic function (Grace et al., under review). Mtb resisters (characterized as highly Mtb exposed individuals that persistently tested negative in both TST and IGRA), similar to health care worker cohorts described above, revealed distinct PPD-specific IgG Fc-glycosylation patterns compared to individuals with LTBI, in which their IgG showed accumulated monogalactosylated (G1) glycans with an increased level of fucosylation and bisecting GlcNAc, but less core sialyation (163). These selectively enriched glycan structures were associated with increased NK cell IFN-γ release facilitated by augmented FcγRIIIa (CD16a) engagement leading to better *in vitro* Mtb control (124, 185). Collectively, Mtb-specific Fc-glycosylation may serve as biomarker to differentiate between ATB and LTBI.

Based on the wide variety of Ab effector functions available, AMI can significantly contribute to protection against Mtb at different stages of infection. Moreover, B-cells and Abs, specifically mucosal IgA, might also be useful biomarkers for vaccine induced protective immunity against Mtb. However, the majority of studies only include quantification of Ab levels, while not taking into account functional assessment of Mtb-specific Abs. Hence, more functional read-outs are needed for B-cells and Abs to evaluate them as correlates of protection and to gain a deeper understanding of the quality of humoral immunity during different stages of Mtb-infection (Figures 1, 2).

## B-cells and Abs as Targets for Vaccination

The majority of designed novel TB-vaccines has concentrated on the induction of CMI (64, 233). Some vaccines, including BCG, induce memory as reflected by the detection of PPD specific memory B-cells (234). However, several recent vaccine studies in mice, NHPs and humans have shown significant induction of Abs against Mtb as well, which might contribute to the vaccine efficacy observed.

Prados-Rosales et al. (235–240) conducted a study with a TB-vaccine that selectively elicits AMI against the tubercle bacillus (241). More specifically, two polysaccharide (PS)-conjugate vaccines were created, by linking the capsular PS AM to either Ag85b or to *Bacillus anthracis* protective Ag. Both PS-conjugate vaccines substantially diminished lung inflammation and mycobacterial dissemination to the spleen in mice challenged with virulent Mtb. More importantly, passive transfer of immune serum derived from AM-immunized mice offered protection, as assessed by decreased mycobacterial loads in both the lungs and spleen when delivered before aerosol Mtb-infection of naïve mice (241). Corroborating these data, mice immunized with a PS-conjugate vaccine of Ag85b and AM generated high AM-specific IgG levels and showed protection against Mtb, reflected by both prolonged survival and diminished lung pathology (239). More recently, booster vaccination with a whole cell inactivated vaccine (heat-killed MTBVAC) increased protection against Mtb after intranasal vaccination of BCG-primed mice in comparison to subcutaneous BCG only (235). This improved protection correlated with induction of PPD-specific Abs in the BAL fluid that opsonized Mtb. These data were supported by similar data obtained in NHPs (235).

In other NHP studies, vaccine-induced local Ab responses correlated with protection against Mtb-infection and disease (212). BCG vaccination was delivered to rhesus macaques either via the standard intradermal route or via endobronchial instillation, a mucosal route of immunization, which resulted in strong protection against subsequent repeated low-dose infection with Mtb, as measured by reduced lung bacillary loads and diminished histopathology. Interestingly, mucosally vaccinated rhesus macaques had high levels of Ag-specific IgA locally in the BAL fluid. Similarly, intravenous administration of BCG in rhesus macaques induced protection against TB and superior Mtb-specific IgG, IgA and IgM responses in BAL fluid and plasma in comparison to intradermal BCG vaccination (213). Together, these studies describe an association between (local) humoral immune responses and protection against Mtb after BCG immunization, putting humoral immunity forward as a potential correlate of protection.

In humans, several TB-vaccine trials showed correlations between specific Abs and vaccine efficacy against Mtb-infection and/or disease. In a large-scale phase 2b efficacy trial in BCG vaccinated South African infants, utilizing the recombinant vaccinia Ankara virus modified to express Ag85A (MVA85A) (242), the presence of Ag-specific IgG titers correlated with a reduced risk of developing TB disease (101). Furthermore, protection was achieved using a subunit vaccine in the M72/ASO1_E_ vaccine trial (243). This clinical phase 2b trial demonstrated that the incidence of ATB disease was significantly reduced in Mtb-infected adults vaccinated with M72/ASO1_E_ relative to placebo. Besides robust activation of T-cells, the vaccine also elicited a strong Ag-specific IgG response that persisted up to at least 36 months post immunization (244, 245). Thus, collectively, the data obtained from mouse, NHP and human vaccination studies support the possibility that AMI might contribute to the protective effects of certain TB-vaccines.

### Preventive and Therapeutic TB-Vaccine Design

An ideal anti-TB-vaccine would protect against both Mtb-infection in exposed individuals and the establishment of disease in already Mtb-infected persons. There is a desperate need for the development of more effective TB-vaccines for children, adolescents and adults. To that end, the emerging appreciation of the role of B-cells and Abs in combatting Mtb (6, 7, 30, 31, 37) in combination with the existence of naturally arising human Abs that are probably functionally protective against Mtb-infection (76), points toward an exciting and promising new path to explore.

BCG as neonatal vaccination against TB has not only reduced the incidence of severe forms of TB in children, but has also reduced all-cause childhood mortality as a result of heterologous protection against non-related pathogens (246–249). BCG vaccinated children had increased levels of Abs in their serum indicating enhanced activation of cell-mediated responses, in particular of humoral immune components (246–249). Although most studies focused on heterologous protection in young children, more recently, it was suggested that also vulnerable elderly could benefit from BCG vaccination to protect against unrelated pathogens (250, 251). BCG did not only enhance responses against natural infections, but also increased the magnitude of humoral responses induced by vaccines against non-related pathogens (246). The mechanism behind these heterologous protective effects is called trained innate immunity and reflects an increased state of responsiveness of mostly innate immune cells due to epigenetic alterations and metabolic rewiring independent of Ag (252). However, it is unknown if similar processes may also alter the response-readiness state of Ag specific adaptive immune players, in particular because the reprogramming occurs in the hematopoietic stem and progenitor compartment (253). Nevertheless, training of innate players by live vaccines, most prominently BCG, will have significant effects on the magnitude of both innate and adaptive responses, including humoral immunity, which will significantly contribute to vaccine induced protection. Novel live vaccines may have a similar training capacity, in particular BCG variants or live attenuated Mtb strains, and thereby might enhance humoral responses, however for other vaccine formulations this remains to be investigated. Prime-boost regimens, including those with a BCG prime, may be superior in activating B-cells partly by enhanced, trained status of key players. A more detailed analysis of these effects for each of the novel vaccine candidates is strongly encouraged.

For the development of a TB-vaccine that elicits not only cellular, but also functional humoral responses, it is of importance to take the route of delivery into consideration (254). Pre-exposure vaccines that aim at preventing infection with Mtb may need to induce IgA production locally in the lungs (255), whereas post-exposure vaccines that aim to either modify or prevent clinical disease in already Mtb-infected individuals most likely will need to trigger a systemic response including IgG (180).

## Discussion

*Mycobacterial tuberculosis* remains a challenging and threatening pathogen, affecting many people globally. Ample epidemiological studies indicate that progression of disease is strongly associated with the immunological competency of the host, and therefore, a more in-depth understanding of the effector anti-TB immune responses is pivotal for the design of next-generation preventive and therapeutic approaches in the fight against this pathogen. The complementary effect of immune components other than classical Th1/Th17 cellular responses, such as Abs, should be considered. Abs and B-cells on their own may not be sufficient to combat Mtb, however there is accumulating evidence that they can complement and enhance CMI. Clinical vaccine evaluation studies should incorporate not only the quantitative assessment of these responses, but also their functional capacity to reduce Mtb burden to assess their future value as correlates of protection and as mediators of protection (Figure 1).

The majority of Ab studies largely concentrated on their utility as diagnostic tools, with little to no attention for dissection of Abs at the isotype and subclass level in relation to TB pathogenesis and resolution (256). However, both Ab isotype, subclass and post-translation modifications might alter functional properties toward Mtb (180). Such differences are most likely a result of structural, antigenic and functional divergences in the Fab and Fc region of Ab isotypes (257). Hence, a more comprehensive insight through detailed immunoprofiling of Mtb-specific Ab responses during disease progression and resolution is essential, not only for TB diagnosis, but also potentially for therapeutic monitoring, and the identification of correlates of protection and markers of disease.

As B-cells and Abs significantly add to the repertoire of effector responses against TB, these two immune components could represent interesting targets for vaccination. However, due to highly heterogenous Ab responses during natural TB infection and disease (258), it might be required to employ strategic vaccine design and specific delivery routes to effectively induce protective rather than enhancing Abs. Mucosal vaccination routes may be more likely to induce protective Mtb specific IgA, however, possibly also other protective vaccines may induce similar mucosal responses, but they have not yet been rigorously assessed. Live vaccines or selected adjuvants may skew strong Ab responses and influence Ab isotype and/or Fc glycosylation profiles (259, 260). Adjuvants characterized by low inflammatory and reactogenic profiles that could be safely administered through the mucosal route may be promising candidates (261).

While the discovery of BCG and antibiotics has been ground-breaking in the prevention and treatment of ATB, major knowledge gaps remain regarding better prevention and treatment of Mtb in both children and adults. Novel host-directed therapeutic and vaccine development efforts will need to go beyond harnessing and improving only cellular immune responses, and should likely also engage the broad range of B-cell and Ab effector functions. As a next step, new tools that probe specificity, affinity, isotype, subclass, function and glycosylation of Abs should be developed. All accumulated evidence so far warrants detailed monitoring of B-cells and Abs in future vaccine efficacy studies, not only as correlates of protection, but also as potential contributors to protection. Since Abs are highly diverse, and this diversity relates to their functional tenures, it is important to not only assess their levels and titers, but also to dissect their functional contribution to mycobacterial inhibition and killing. Even though B-cells and Abs may not be fully sufficient on their own, they clearly can contribute to the total effector response and thereby provide novel and important targets for future studies and interventions against Mtb and other intracellular pathogens.

## Author Contributions

WR, TO, and SJ drafted the original manuscript outline, composed the initial draft and corrected the manuscript to its final version. All authors contributed to the article and approved the submitted version.

## Conflict of Interest

The authors declare that the research was conducted in the absence of any commercial or financial relationships that could be construed as a potential conflict of interest.

## Abbreviations

Ab: antibodies
Acr: α-crystallin
ADCC: antibody-dependent cell-mediated cytotoxicity
ADCP: antibody-dependent cellular phagocytosis
Ag: antigen
AM: arabinomannan
AMI: antibody-mediated immunity
AP: activator protein
APC: antigen-presenting cell
ATB: active tuberculosis disease
BAL: bronchoalveolar lavage
BCF: B-cell follicle
BCG: Bacillus Calmette-Guérin
Breg: regulatory B-cell
C1q: Complement component 1q
Ca^2+^: calcium
CFP-10: 10 kDa culture filtrate protein
CMI: cell-mediated immunity
CVID: common variable immunodeficiency
CXCL: CXC chemokine ligand
CXCR: CXC chemokine receptor
ESAT-6: early secreted Ag of 6 kDa
Fab: Ag-binding fragment
FASL: Fas ligand
Fc: crystallizable fragment
FcγRI: Fc gamma receptor I
FcR: Fc receptor
GC: germinal center
HBHA: heparin-binding hemagglutinin adhesin
HIV: human immunodeficiency virus
iBALT: inducible bronchus-associated lymphoid tissue
IFN: interferon
Ig: immunoglobulin
IGRA: IFN-γ release assay
iNOS: inducible nitric oxide synthase
IRF: interferon regulatory factor
IVIg: intravenous immunoglobulin
LAM: lipoarabinomannan
LAMP-1: lysosomal-associated membrane protein 1
LTBI: latent tuberculosis infection
mAb: monoclonal antibody
ManLAM: mannose-capped lipoarabinomannan
Mtb: *Mycobacterium tuberculosis*
MVA85A: Ankara virus modified to express Ag85A
NF-κB: nuclear factor kappa-light-chain-enhancer of activated B-cells
NHP: non-human primate
NK: natural killer
pIgR: polymeric immunoglobulin receptor
PIM: phosphatidyl-myo-inositol mannoside
PPD: protein purified derivative
PS: polysaccharide
QFN: QuantiFERON-TB Gold In-Tube test
QFT: QuantiferonTB Gold
SCID: severe combined immunodeficient
sIgA: secretory IgA
TB: tuberculosis
Th: T helper
TLR: Toll-like receptor
TNF: tumor necrosis factor
TRIM21: FcR tripartite motif-containing protein 21
TST: tuberculin skin test
WT: wild type
XLA: X-linked agammaglobulinemia.
